# Glycyrrhizin Protects Mice Against Experimental Autoimmune Encephalomyelitis by Inhibiting High-Mobility Group Box 1 (HMGB1) Expression and Neuronal HMGB1 Release

**DOI:** 10.3389/fimmu.2018.01518

**Published:** 2018-07-02

**Authors:** Yan Sun, Huoying Chen, Jiapei Dai, Zhongjun Wan, Ping Xiong, Yong Xu, Zhengrong Han, Weitai Chai, Feili Gong, Fang Zheng

**Affiliations:** ^1^Wuhan Institute for Neuroscience and Neuroengineering, South-Central University for Nationalities, Wuhan, China; ^2^Department of Immunology, School of Basic Medicine, Tongji Medical College, Huazhong University of Science and Technology, Wuhan, China; ^3^Department of Neurobiology, College of Life Sciences, South-Central University for Nationalities, Wuhan, China; ^4^Department of Laboratory Medicine, The Second Affiliated Hospital of Guilin Medical University, Guilin, China; ^5^Key Laboratory of Organ Transplantation, Ministry of Education, Chinese Academy of Medical Sciences, Wuhan, China; ^6^NHC Key Laboratory of Organ Transplantation, Chinese Academy of Medical Sciences, Wuhan, China; ^7^Key Laboratory of Organ Transplantation, Chinese Academy of Medical Sciences, Wuhan, China

**Keywords:** high-mobility group box 1, experimental autoimmune encephalomyelitis, multiple sclerosis, glycyrrhizin, neurons

## Abstract

The inflammatory mediator high-mobility group box 1 (HMGB1) plays a critical role in the pathogenesis of human multiple sclerosis (MS) and mouse experimental autoimmune encephalomyelitis (EAE). Glycyrrhizin (GL), a glycoconjugated triterpene extracted from licorice root, has the ability to inhibit the functions of HMGB1; however, GL’s function against EAE has not been thoroughly characterized to date. To determine the benefit of GL as a modulator of neuroinflammation, we used an *in vivo* study to examine GL’s effect on EAE along with primary cultured cortical neurons to study the GL effect on HMGB1 release. Treatment of EAE mice with GL from onset to the peak stage of disease resulted in marked attenuation of EAE severity, reduced inflammatory cell infiltration and demyelination, decreased tumor necrosis factor-alpha (TNF-α), IFN-γ, IL-17A, IL-6, and transforming growth factor-beta 1, and increased IL-4 both in serum and spinal cord homogenate. Moreover, HMGB1 levels in different body fluids were reduced, accompanied by a decrease in neuronal damage, activated astrocytes and microglia, as well as HMGB1-positive astrocytes and microglia. GL significantly reversed HMGB1 release into the medium induced by TNF-α stimulation in primary cultured cortical neurons. Taken together, the results indicate that GL has a strong neuroprotective effect on EAE mice by reducing HMGB1 expression and release and thus can be used to treat central nervous system inflammatory diseases, such as MS.

## Introduction

Multiple sclerosis (MS) is an autoimmune disease characterized by inflammation, demyelination, and neurodegeneration in the central nervous system (CNS) (1). Experimental autoimmune encephalomyelitis (EAE) is a mouse model for improving understanding and treatment of MS (2). Although several medications, such as alemtuzumab, rituximab, and fingolimod, have been used to prevent MS relapses (3), therapies for acute MS exacerbations are still limited. Several studies have shown that the inflammatory cascade accompanied by myelin-specific CD4^+^ T helper cell-mediated autoimmunity is un-ignorable for the pathogenesis of MS (4, 5). Thus, efforts to achieve a better understanding of the mechanisms controlling inflammation might be a strategy for the treatment of MS.

Extensive work has cast light on the role of high-mobility group box 1 (HMGB1), an inflammatory mediator, in the pathogenesis of various neurological diseases, including Alzheimer’s disease (6), Huntington’s disease (7), Parkinson’s disease (8), and epilepsy (9). Recently, it has been demonstrated that HMGB1 has a detrimental effect during the pathogenesis of MS. Increased HMGB1 levels have been found in the cerebrospinal fluid (CSF) and white matter (WM) areas of MS patients (10), indicating that HMGB1 is upregulated and released into the CNS during MS. At the site of inflammation and/or damage, nuclear HMGB1 can be translocated from the nucleus to the cytoplasm and/or the extracellular milieu by passive release or active secretion, and it acts as a damage-associated molecular pattern signal (11). The released HMGB1 targets its receptor, advanced glycation end product (RAGE), or a toll-like receptor (TLR) such as TLR-2/4, and induces the expression of pro-inflammatory cytokines including tumor necrosis factor-alpha (TNF-α), IL-6, and IL-1β, as well as leukocyte adhesion molecules and chemokines (12).

In addition to passive release by necrotic cells, HMGB1 is also actively secreted by innate immune cells such as monocytes and macrophages (13, 14). A mechanism of HMGB1 secretion from living cells has been described (15–17). Previous studies have shown that neurons and glial cells can also secrete HMGB1 (18, 19). However, it is unclear whether CNS-derived cells or other infiltrating cells release HMGB1 during MS/EAE. Previously, we have revealed elevated HMGB1 levels in the serum, CSF, and spinal cord homogenate during EAE progression correlating with disease severity (20), suggesting that resident cells or infiltrating cells in the CNS are likely to release HMGB1 during EAE. Moreover, systemic treatment with neutralizing HMGB1 antibody markedly ameliorates mouse EAE (20–22). We have also confirmed that blockade of CNS local HMGB1 with anti-HMGB1 monoclonal antibody administered intracerebroventricularly clearly suppresses the progression of EAE (20). These studies suggest that HMGB1 plays a detrimental role in the pathophysiology of EAE and that inhibition of HMGB1 release may be a potential approach for anti-inflammatory therapy in MS/EAE.

Glycyrrhizin (GL) is the chief bioactive component of licorice root extract and is found as an additional HMGB1 inhibitor. GL is a natural anti-inflammatory product compared to anti-HMGB1 monoclonal antibody; GL also has pleiotropic pharmacologic activities, such as anti-tumor, anti-viral, hepatoprotective, neuroprotective properties, and anti-inflammatory effects. Furthermore, it has functions of liver protection and membrane stabilization, which are clinically important in the treatment of hepatitis B and C in Japan (23). More importantly, recent studies have shown that GL provides protection in CNS diseases, such as focal ischemia (24), brain injury (25), and Parkinson’s disease (26). Previous studies have suggested that GL binds directly to both HMG boxes in HMGB1, thereby conferring its neuroprotective effects (27, 28). However, the therapeutic effect of GL treatment on EAE has not been confirmed.

In the present study, we used the HMGB1 inhibitor GL in the EAE mouse model to demonstrate that GL can attenuate the severity of EAE by reducing HMGB1 levels in different body fluids, inhibiting neuronal damage, inactivating astrocytes and microglia, and reducing the number of HMGB1-positive astrocytes and microglia. In addition, GL reverses the release of HMGB1 into the medium induced by TNF-α stimulation in primary cultured cortical neurons. Our data indicate a potential therapeutic effect of GL against MS.

## Materials and Methods

### Mice

Specific pathogen-free female C57BL/6 mice (5–6 weeks) were purchased from Shanghai SLAC Laboratory Animal Co. Ltd. (Shanghai, China), randomly assigned to different experimental groups, and maintained in Micro-Isolator cages with standard laboratory chow and water. All animal experimental procedures were approved by the Animal Care and Use Committee of Tongji Medical College of Huazhong University of Science and Technology.

### Active EAE Induction and Assessment

The EAE animal model was induced as previously described (20, 29). Mice were evaluated daily according to their clinical manifestations of the disease: 0, no symptoms; 0.5, weak tail; 1, floppy tail; 2, hind limb weakness; 2.5, one hind limb paralyzed; 3, both hind limbs paralyzed; 3.5, forelimb weakness and hindlimb paralysis; 4, both hind limb and forelimb paralysis; and 5, moribund state or death. Animal disease severity was scored daily.

### GL Administration

Glycyrrhizin (Minophagen Pharmaceutical Co., Tokyo, Japan) was dissolved in 50 mM NaOH at 37°C, and the pH was adjusted to 7.4 using 0.5 M Tris–HCl buffer (pH 6.8). Mice received intraperitoneal (i.p.) injections with three different concentrations of GL (10, 25, and 50 mg/kg) every other day from days −1 to 11 (strategy I), from days 12 to 22 (strategy II), or from days 15 to 23 (strategy III) after induction of EAE. No mice became moribund in the study. Animals were monitored daily throughout the whole experiment.

### Tissue Preparation and Histological Analysis

Twenty-five days after immunization with MOG_35–55_, the thoracic spinal cord tissues were prepared as previously described (20, 29) and sectioned at a 20-µm thickness following histological, immunohistochemistry, and immunofluorescent staining. For histological evaluation, sections were stained with hematoxylin and eosin (H&E; Sigma-Aldrich, St. Louis, MO, USA) and Luxol Fast Blue (LFB; Sigma-Aldrich) following standard protocols for conventional light microscopy. Cell infiltration was scored as follows based on H&E staining: 0, no inflammation; 1, a few scattered inflammatory cells; 2, cellular infiltrates around blood vessels and meninges; 3, moderate cellular infiltrates in parenchyma, less than 50% of the WM areas occupied; 4, extensive cellular infiltrates in parenchyma, more than 50% of the areas occupied. Demyelination was scored as follows based on LFB: 0, no demyelination; 1, slight subpial demyelination; 2, a few areas occupied of demyelination; 3, integration of the myelin sheath around the vascular or subpial; 4, moderate areas of demyelination involving less than 50% of the WM areas occupied; and 5, diffuse demyelination involving more than 50% of the WM in the presence of cellular infiltrates in the CNS parenchyma.

### Immunohistochemical Staining

For immunohistochemistry, frozen sections were stained with anti-HMGB1 (1:800; Abcam, Cambridge, MA, USA), anti-glial fibrillary acidic protein (GFAP, 1:1,000; Abcam), anti-NeuN (1:100; Abcam or 1:200; Millipore, Bedford, MA, USA), anti-Iba1 (ionized calcium binding adaptor molecule 1, 1:600; Abcam), and anti-CD3 (1:50; Abcam) antibodies as previously described (18). Quantification of HMGB1-positive (HMGB1^+^) cells in the total section, WM, or gray matter (GM) were examined in each section under 40× magnification using a motorized microscope (ECLIPSE 90i, Nikon, Japan) and with Image-Pro Plus 6.0 software (Media Cybernetics, Rockville, MD, USA). The number of GFAP-positive (GFAP^+^) cells and Iba1-positive (Iba^+^) cells in the WM or GM was counted in a fixed area in six different sections per mouse, while the number of NeuN-positive (NeuN^+^) cells was counted in the whole GM in six different sections per mouse.

### Immunofluorescence Staining

For immunofluorescence, endogenous peroxidase and biotin were blocked with 5% BSA, and then the sections were incubated overnight at 4°C with the primary antibodies mentioned above. After thorough washing, the sections were incubated with fluorescein-conjugated secondary antibodies for 1 h at 37°C. Secondary antibodies included the following: mouse or rabbit Alexa Fluor 488 or Alexa Fluor 568 (1:50; CWBIO, Beijing, China). The sections were then mounted in DAPI (Invitrogen, Carlsbad, CA, USA) for nuclear staining. All cells were examined using a laser scanning confocal microscope (FV-500, Olympus Inc., Tokyo, Japan), and images were acquired using an FV10-ASW 2.1 image visualization system (Olympus). For cell counting, 12 fields in each spinal cord section from four mice (three or four sections per mouse) per group were determined. The data are presented as the number of positive cells per square millimeter.

### CSFs and Serum Collection

Both CSF and serum samples were collected as previously described (20), snap-frozen in liquid nitrogen, and then stored at −80°C until use.

### Spinal Cord Homogenate

Fresh thoracolumbar spinal cord of each mouse was obtained on day 25, weighed and then rapidly homogenized with PBS (100 mg tissue per 1 mL of 0.01 M PBS) containing a protease inhibitor cocktail. The sample lysates were centrifuged at 2,000 rpm for 5 min at 4°C. The supernatant was collected to measure HMGB1, TNF-α, IFN-γ, IL-17A, IL-4, IL-1β, IL-6, and transforming growth factor-beta 1 (TGF-β1) by ELISA. Cytokines are expressed as picogram per gram of total protein.

### Extraction of Total Protein

Fresh thoracolumbar spinal cords were collected and homogenized on ice in RIPA buffer (150 mM NaCl, 50 mM Tris–HCl, 1% TritonX-100, 0.02% sodium azide, and proteinase inhibitor cocktail, pH 7.4). After centrifugation, the supernatant was collected and analyzed for Western blotting.

### Western Blotting

Protein extraction and quantification were performed following the manufacturer’s instructions for the reagents used. Each sample containing 40 µg of protein was resolved by 12% SDS-PAGE, and the samples were then electrophoretically transferred onto Immuno-Blot polyvinylidene fluoride membranes (Hybond Inc., Escondido, CA, USA). The membranes were blocked with 5% non-fat dry milk in TBS solution for 1 h at room temperature, followed by incubation overnight with primary antibodies, including rabbit anti-HMGB1 (1:8,000; Abcam), mouse anti-β-actin (1:500; Santa Cruz Biotechnology, Dallas, TX, USA), and rabbit anti-Lamin B1 (1:500; Proteintech Group, Inc., Chicago, IL, USA) antibodies. The membrane was then incubated with a horseradish peroxidase-conjugated anti-IgG (1:2,000; CWBIO) for 1 h at room temperature. Subsequently, immunoreactive bands were visualized using an ECL kit (Beyotime Institute Biotech, Shanghai, China) and quantified by densitometry using the ECL-Plus detection system (ClinX Sciences Instruments, Shanghai, China). β-actin was used as controls for protein loading.

### Primary Neuronal Cultures

Primary neuronal cultures were prepared from cerebral cortices of 1- to 2-day-old neonatal C57BL/6 mice as previously described (29). Half of the old medium was replaced every 3 days, and neuronal confluency was achieved and performed for immunophenotyping on the seventh day after seeding. In this system, more than 98% of the cells were identified as neurons by anti-NeuN antibody staining.

### TNF-α Treatment of Primary Cultured Neurons

Neurons were seeded in 6-well culture plates at a density of 1 × 10^6^ cells/well and cultured 7 days after seeding to allow the cells to reach confluency. Primary cultured neurons were then treated with TNF-α (200 ng/mL; R&D Systems, Minneapolis, MN, USA) in the presence or absence of GL (3 mmol/L) for 18 h. Culture medium was collected and concentrated as described previously for Western blot analysis and ELISA (29). The survival rate of neurons was determined using the 0.4% Trypan blue exclusion method.

### ELISA

Two mouse HMGB1 ELISA kits (Uscn Life Science Inc., Wuhan, China; CUSABIO, Houston, TX, USA) were used to measure the levels of HMGB1 in serum, CSF, spinal cord homogenate, and culture medium according to the manufacturers’ protocol. The levels of mouse TNF-α, IL-6, IFN-γ, IL-17A, IL-4, IL-1β, and TGF-β1 in serum, spinal cord homogenate, and culture medium were also measured by ELISA (ELISA kits: TNF-α and IL-6, BioLegend, San Diego, CA, USA; IFN-γ and IL-17A, San Diego, CA, USA; IL-4, IL-1β, and TGF-β1, R&D Systems, Minneapolis, MN, USA). Cytokine concentrations were determined using the relevant standard curves.

### Statistical Analysis

Experimental data were analyzed using GraphPad Prism 5.0 (GraphPad Software Inc., San Diego, CA, USA). Experimental data are presented as the mean ± SEM. Comparisons between groups were analyzed using a two-tailed Student’s *t*-test, one-way analysis of variance followed by Bonferroni correction for measurement data, or Mann–Whitney *U* test for ordinal data, as appropriate. A value of *P* < 0.05 was considered statistically significant.

## Results

### GL Treatment Attenuated the Progression of EAE

**Figure 1:**
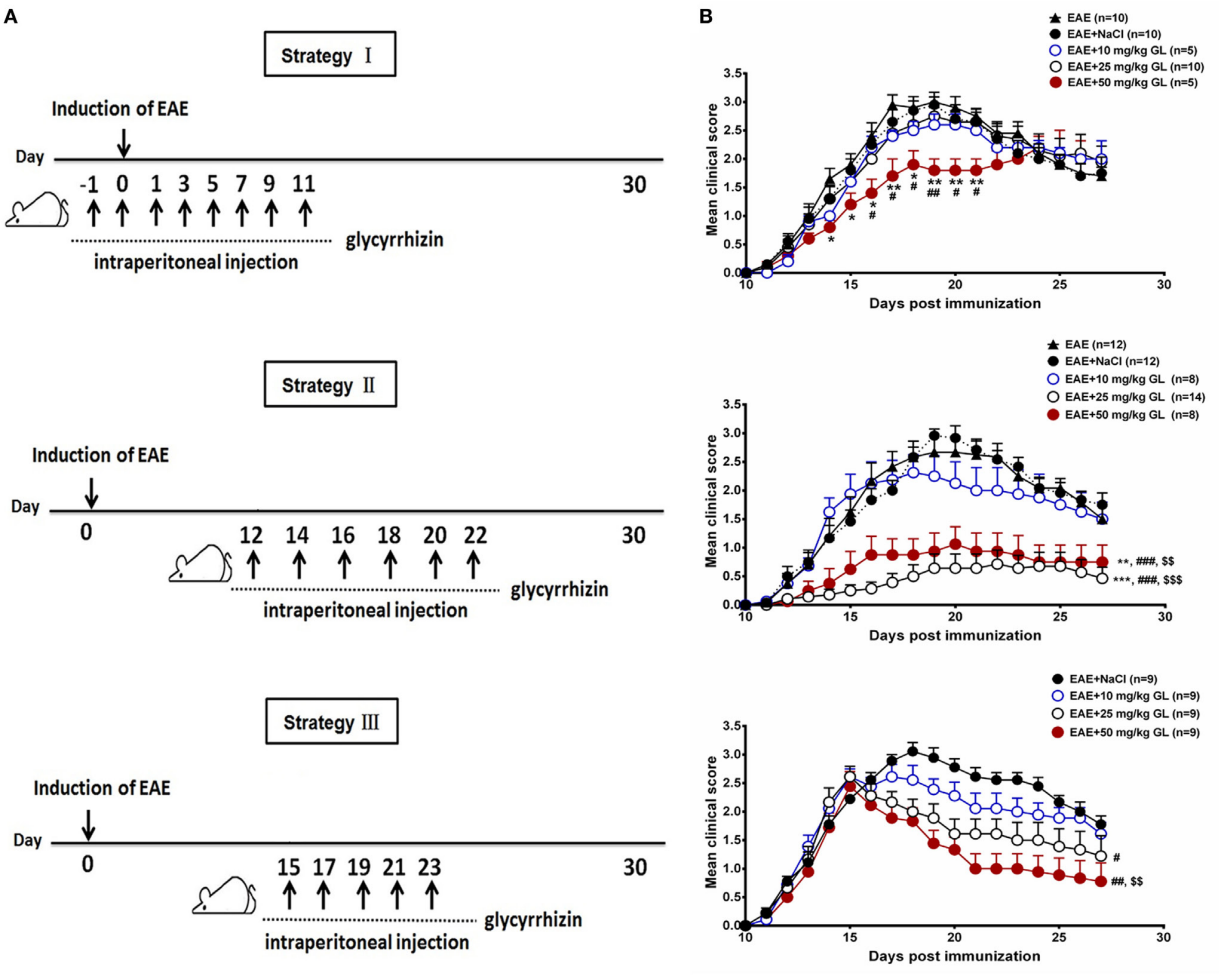
The effects of glycyrrhizin (GL) on the development of experimental autoimmune encephalomyelitis (EAE). **(A)** Schematic workflow of GL treatment. Mice were injected i.p. with 10, 25, or 50 mg/kg GL or 0.9% NaCl each time. **(B)** The effects of different concentrations of GL treatment on disease score. Mice were injected i.p. from days −1 to 11 (strategy I), days 12 to 22 (strategy II), or days 15 to 23 (strategy III) with GL or NaCl. *P < 0.05, **P < 0.01, ***P < 0.001 vs EAE group; ^#^*P* < 0.05, ^##^*P* < 0.01, ^###^*P* < 0.001 vs EAE + NaCl group, ^$$^*P* < 0.01, ^$$$^*P* < 0.001 vs EAE + 10 mg/kg GL group, using the Mann–Whitney *U* test for the time point or cumulative disease score. The results shown represent one experiment or two independent experiments.

To determine the effects of GL on the initiation and progression of EAE, mice were treated using three different strategies with three different concentrations of GL (10, 25, and 50 mg/kg) as shown in Figure 1A. Similar treatment strategies have been reported by our and other research groups (20, 30, 31). Strategy I injection occurred from days −1 to day 11, which we refer to as early GL treatment; strategy II injection was from days 12 to 22 as the midterm-late GL treatment; strategy III injection was from days 15 to 23 as the late GL treatment (Figure 1A). For early GL treatment, we found that 10 and 25 mg/kg GL had no significant influence on EAE incidence, mean maximal score or disease onset, while 50 mg/kg GL attenuated the disease severity from days 15 to 21 (Figure 1B, upper panel; Table 1). However, the protective effect disappeared at the late stage of EAE. For midterm-late and late GL treatment, both 25 and 50 mg/kg GL significantly attenuated the disease severity and had long-term protective effects in EAE mice (Figure 1B; Table 1). EAE mice began to develop clinical symptoms around day 12 in our laboratory, but usually only 1–2 or 0 mice in each group developed clear signs on this day. Thus, treatment with a sufficient dose of GL from day 12 can affect the incidence and onset time of midterm-late GL-treated EAE mice. As shown in Table 1, midterm-late treatment with 25 and 50 mg/kg GL clearly decreased the disease incidence and delayed the onset of EAE. Mice that did not develop symptoms showed a delayed onset or even were no longer affected, and the severity of the mice that had developed symptoms was attenuated. The results indicate that GL treatment confers the therapeutic effects in EAE mice.

**Table 1:** Clinical features of experimental autoimmune encephalomyelitis (EAE) mice with glycyrrhizin (GL) treatment.

	Strategy I: pre-onset stage				Strategy II: onset and peak stages				Strategy III: peak stage			
Group	No. with EAE/Total	Incidence	Mean maximal score (mean ± SEM)	Disease onset (day, mean ± SEM)	No. with EAE/Total	Incidence	Mean maximal score (mean ± SEM)	Disease onset (day, mean ± SEM)	No. with EAE/Total	Incidence	Mean maximal score (mean ± SEM)	Disease onset (day, mean ± SEM)
EAE	10/10	100	3.15 ± 0.15	12.30 ± 0.37	12/12	100	2.96 ± 0.20	13.17 ± 0.36				
EAE + NaCI	10/10	100	3.05 ± 0.13	12.10 ± 0.31	12/12	100	3.04 ± 0.16	12.92 ± 0.42	9/9	100	3.17 ± 0.15	11.56 ± 0.18
EAE + 10 mg/kg GL	5/5	100	2.70 ± 0.20	12.60 ± 0.24	8/8	100	2.38 ± 0.32	12.38 ± 0.32	9/9	100	2.78 ± 0.19	11.78 ± 0.15
EAE + 25 mg/kg GL	10/10	100	2.90 ± 0.15	12.50 ± 0.40	7/14	50	0.86 ± 0.27^***, ###, $$^	15.71 ± 1.23^*, #, $^	9/9	100	2.61 ± 0.18^#^	11.56 ± 0.18
EAE + 50 mg/kg GL	5/5	100	2.30 ± 0.20^*, #^	12.20 ± 0.37	6/8	75	1.19 ± 0.33^**, ###, $^	14.50 ± 0.67^#, $^	9/9	100	2.44 ± 0.26^#^	12.00 ± 0.24

“EAE”: EAE induction without treatment. “EAE + NaCl”: EAE induction with 0.9% NaCl treatment. “EAE + GL”: EAE induction with 10, 25, or 50 mg/kg GL treatment. *P < 0.05, ***P < 0.001 vs EAE group; ^#^P < 0.05, ^###^P < 0.001 vs EAE + NaCl group, ^$^P < 0.05, ^$$^P < 0.01 vs EAE + 10 mg/kg GL group, using Mann–Whitney U test. The results shown are one experiment or representative of two independent experiments.

### GL Treatment Decreased Inflammation and Demyelination and Modulated the Secretion of Cytokines in the CNS of EAE

**Figure 2:**
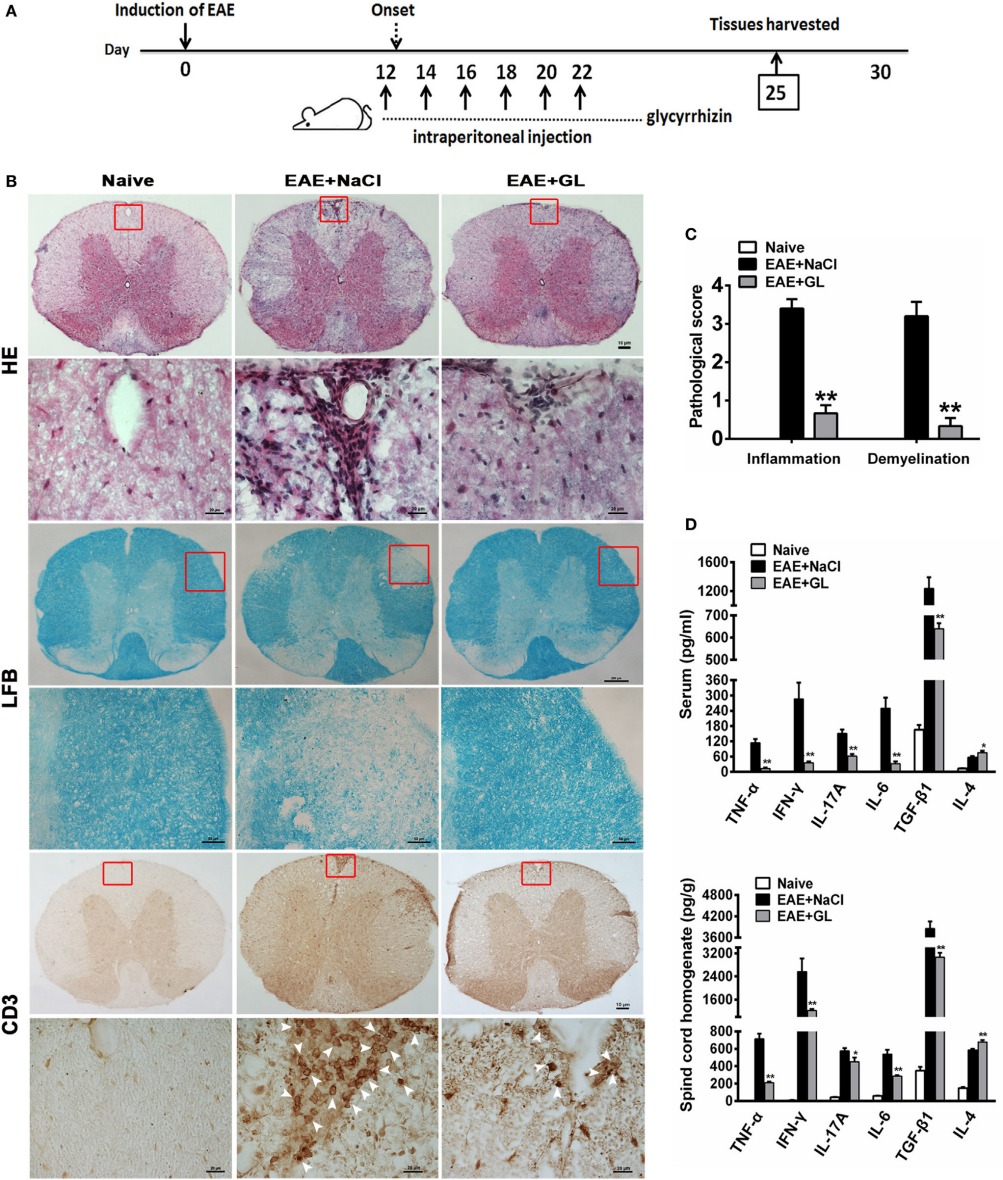
Reduced inflammation and demyelination and modulated inflammation-related cytokines in EAE mice treated with GL. **(A)** Schematic workflow of GL treatment. Mice were injected i.p. with GL (25 mg/kg each time) or 0.9% NaCl every other day from days 12 to 22 post-EAE induction. **(B)** The spinal cord tissues of naive mice and EAE mice treated with NaCl or GL were harvested 25 days after immunization, fixed, sectioned, and stained with H&E (top), LFB (middle), or immunohistochemical staining for CD3 (bottom). For H&E and CD3 staining, scale bars were 10 µm for low magnification and 20 µm for high magnification. For LFB staining, scale bars were 200 µm for low magnification and 50 µm for high magnification. **(C)** Pathological scores for inflammation and demyelination were evaluated based on H&E and LFB staining, respectively. Histology was performed using at least six serial thoracic spinal cord sections in five different mice for each group, and representative images are shown. The data are expressed as the mean ± SEM, **P < 0.05 vs the EAE + NaCl group using the Mann–Whitney *U* test. **(D)** The concentrations of TNF-α, IFN-γ, IL-17A, IL-4, IL-6, and TGF-β1 both in serum and spinal cord homogenate were measured by ELISA at day 25 post-immunization. Data are shown as the mean ± SEM, *n* = 8–12 for serum and *n* = 8 for spinal cord homogenate in each experiment. *P < 0.05, **P < 0.01 vs EAE + NaCl group using the two-tailed Student’s *t*-test. Abbreviations: TNF-α, tumor necrosis factor-alpha; TGF-β1, transforming growth factor-beta 1; EAE, experimental autoimmune encephalomyelitis; GL, glycyrrhizin; H&E, hematoxylin and eosin; LFB, Luxol Fast Blue.

Next, we assessed the effects of GL on the development of EAE. As 25 mg/kg midterm-late GL treatment conferred therapeutic effects on EAE based on the results in Figure 1 and Table 1, we selected the 25 mg/kg midterm-late GL treatment for our subsequent experiments. Either GL or NaCl was injected i.p. into each mouse every other day from days 12 to 22, and spinal cord tissues were harvested from treated mice at day 25 post-immunization (Figure 2A). Histopathological examination of the spinal cord tissue sections from naive mice showed an intact myelin sheath and no mononuclear cell infiltration (Figure 2B, left panel), whereas typical demyelination and inflammation were observed in the EAE + NaCl group (Figure 2B, middle panel). Treatment with GL remarkably reduced CNS demyelination and inflammation in the WM compared to the EAE + NaCl group. In addition, infiltration of CD3^+^ T lymphocytes was substantially decreased in response to GL treatment (Figure 2B, right panel). The pathological scores were also significantly ameliorated (*P* < 0.01 for the infiltration score and demyelination score, respectively) by injection of GL (Figure 2C). Inflammation-related cytokines such as TNF-α, IL-6, IL-4, and TGF-β1, which play a contributory role in EAE pathogenesis, were secreted at significant levels during EAE progression (Figure S1 in Supplementary Material). Here, we found that both in serum and spinal cord homogenate, the levels of TNF-α, IFN-γ, IL-17A, IL-6, and TGF-β1 declined in GL-treated EAE mice, but GL treatment could significantly increase IL-4 secretion (Figure 2D). These results indicate that GL treatment prevents further neuroinflammation of EAE.

### GL Treatment Suppressed the Activation of Astrocytes and Microglia and the Damage to Neurons

**Figure 3:**
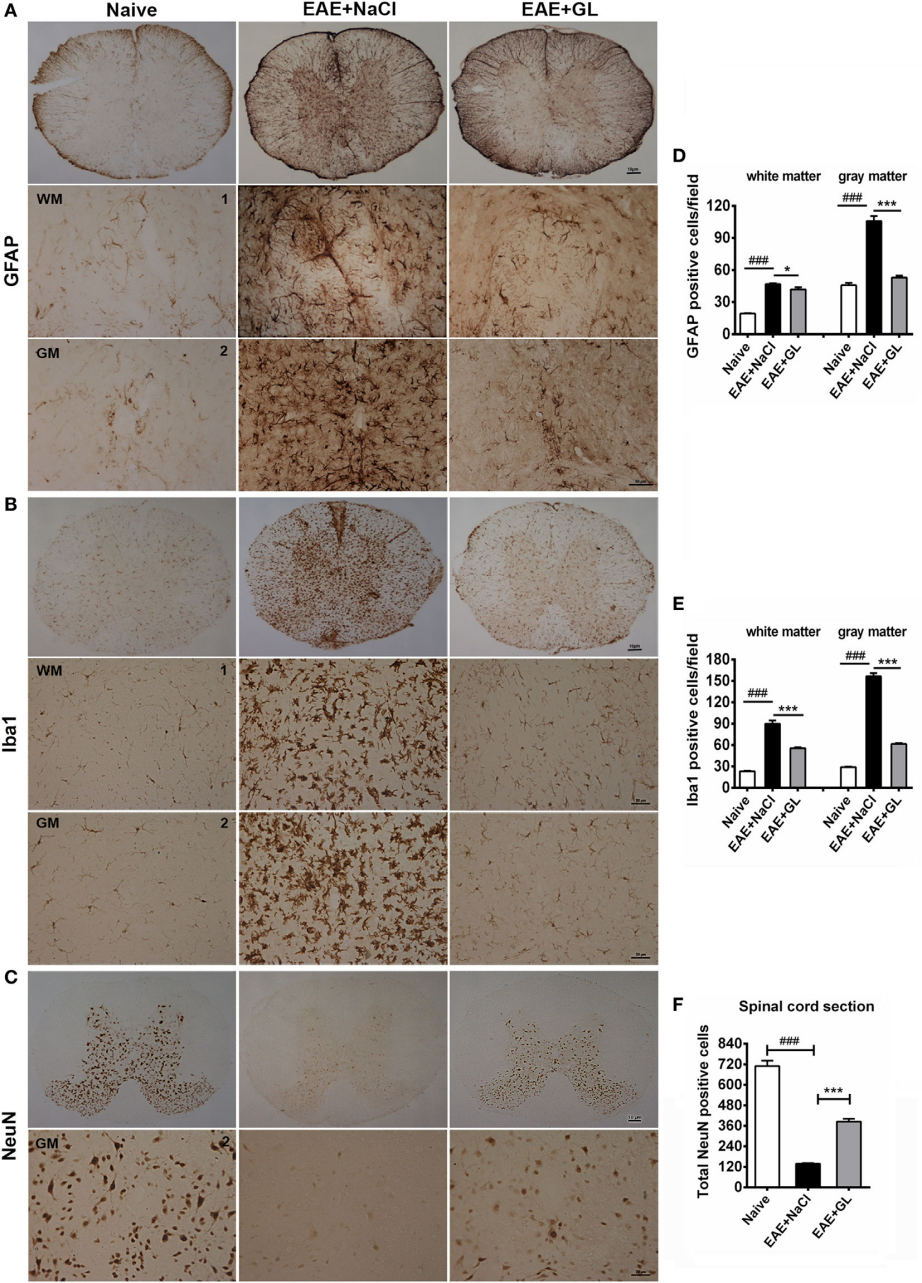
Activated astrocytes, microglia, and damaged neurons in the spinal cord of experimental autoimmune encephalomyelitis (EAE) mice treated with glycyrrhizin (GL). Immunohistochemical staining for glial fibrillary acidic protein (GFAP), Iba1, and NeuN in thoracic spinal cord sections of naive mice and EAE mice treated with either NaCl or GL obtained at day 25 post-immunization. 1: dorsal column; 2: central canal. **(A)** GFAP-positive cells. Scale bars were 10 µm for low magnification and 50 µm for high magnification. **(B)** Iba1-positive cells. Scale bars were 10 µm for low magnification and 20 µm for high magnification. **(C)** NeuN-positive cells. Scale bars were 10 µm for low magnification and 20 µm for high magnification. **(D–F)** The number of GFAP-positive cells, Iba1-positive cells, and NeuN-positive cells in spinal cords. At least six serial thoracic spinal cord sections were analyzed from each mouse, and six mice were included in each group. Data are shown as the mean ± SEM. ^###^*P* < 0.001 vs naive group; *P < 0.05, ***P < 0.001 vs EAE + NaCl group, statistical significance (*P*) was determined by one-way ANOVA with Bonferroni’s test.

Astrocytes and microglia are the resident cells in the CNS. It has been reported that astrocytes and microglia participate in the inflammatory process, leading to the onset of EAE (32, 33). We used GFAP (marker for astrocytes) and Iba1 (marker for microglia) immunostaining to evaluate whether GL could suppress the activation of astrocytes and microglia in the EAE from strategy II treatment mice. GFAP and Iba1 were slightly stained in the spinal cord of naive mice. The GFAP^+^ and Iba1^+^ cells had small cell bodies and long, thin ramified processes (Figures 3A,B, left panels). In the EAE + NaCl group, the astrocytes and microglia became activated, more GFAP and Iba1 were observed (Figures 3A,B, middle panels), and the number of GFAP^+^ and Iba1^+^ cells was significantly increased in the WM and GM of the spinal cord compared with the naive group (Figures 3D,E). In the EAE + GL group, the number of GFAP^+^ and Iba1^+^ cells was significantly decreased compared with the EAE + NaCl group (Figures 3A,B, right panels and Figures 3D,E). In addition, the damage to neurons was observed as smeared NeuN staining, and the number of NeuN^+^ neurons in the whole GM of the spinal cord was significantly decreased in EAE mice treated with NaCl compared with naive mice (Figure 3C, left and middle panels). Following treatment with GL, the number of NeuN^+^ neurons was significantly recovered (Figure 3F). These data suggest that GL treatment alleviated EAE by blocking both gliosis (GFAP-positive cells) and microglial cell activation (Iba1-positive cells) and by providing protection against neuronal damage in the spinal cord.

### GL Treatment Attenuated the Release and Expression of HMGB1 in EAE

**Figure 4:**
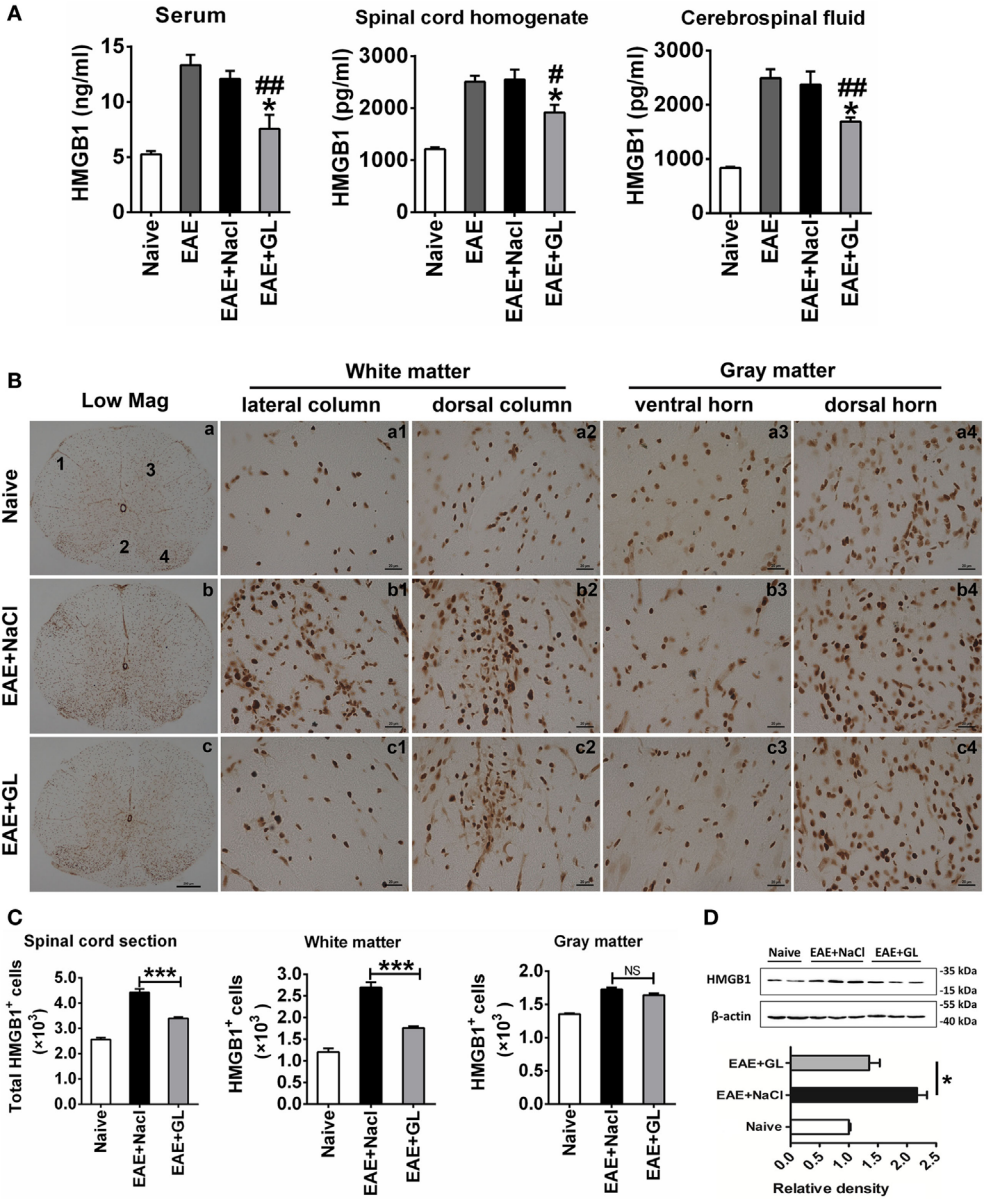
The expression and release of high-mobility group box 1 (HMGB1) inhibited by glycyrrhizin (GL) treatment. **(A)** HMGB1 levels in different body fluids. “Naive,” “experimental autoimmune encephalomyelitis (EAE) + NaCl” and “EAE + GL” mean control group, EAE induction with NaCl treatment group, and EAE induction with GL treatment group, respectively. Data are shown as the mean ± SEM, *n* = 5–8 for serum and cerebrospinal fluid and *n* = 6 for spinal cord homogenate in each experimental group. *P < 0.05 vs EAE + NaCl group, ^#^*P* < 0.05, ^##^*P* < 0.01 vs EAE group, using one-way ANOVA followed by Bonferroni’s test. **(B)** Expression of HMGB1 in thoracic spinal cord sections in naive mice and EAE mice after day 25 treated with either NaCl or GL. 1: lateral column; 2: dorsal column; 3: ventral horn; 4: dorsal horn. Scale bars were 200 µm for low magnification and 20 µm for high magnification. **(C)** The number of HGMB1-positive cells in one spinal cord section. At least six serial thoracic spinal cord sections were analyzed from each mouse, and six mice were included in each group. Data are expressed as the mean ± SEM. ***P < 0.001 vs EAE + NaCl group; NS, not significant, using one-way ANOVA followed by Bonferroni’s test. **(D)** Relative densitometry analysis of HMGB1 (25 kDa) normalized to β-actin (43 kDa). Data are shown as the mean ± SEM, *n* = 4 animals in each experimental group. *P < 0.05 vs EAE + NaCl group, using one-way ANOVA followed by Bonferroni’s test. These data were representative of three independent experiments.

Based on our previous finding (20) that the expression of HMGB1 increased during the progression of EAE (also shown in Figure S1 in Supplementary Material), we investigated the effect of GL administration on the levels of HMGB1 in different body fluids. We used ELISA to measure HMGB1 levels in serum, spinal cord homogenate, and CSF at day 25 post-immunization from midterm-late NaCl or GL-treated EAE mice (strategy II). Higher levels of HMGB1 were detected in serum, spinal cord homogenate, and CSF of the EAE compared with the naive group. However, i.p. injection of GL significantly inhibited the increase in HMGB1 in the three kinds of body fluids of EAE mice (Figure 4A). Moreover, immunohistochemical staining showed that HMGB1 was weakly expressed in the naive mice, but it was increased in the lateral column, dorsal column, ventral horn, and dorsal horn of NaCl-treated EAE mice. Compared with NaCl-treated EAE mice, the expression of HMGB1 was significantly downregulated in GL-treated EAE mice (Figure 4B). Quantitative analysis of HMGB1^+^ cells in total sections, WM, or GM showed that the number of HMGB1^+^ cells was decreased in the GL-treated mice, especially in the WM (Figure 4C). Consistently, the amount of total HMGB1 protein was significantly reduced in the spinal cord of GL-treated EAE compared with NaCl-treated EAE mice (Figure 4D). These data demonstrate that GL treatment reduced the release and expression of HMGB1 in the spinal cord of EAE mice.

### GL Treatment Decreased HMGB1 Expression in Astrocytes and Microglia

**Figure 5:**
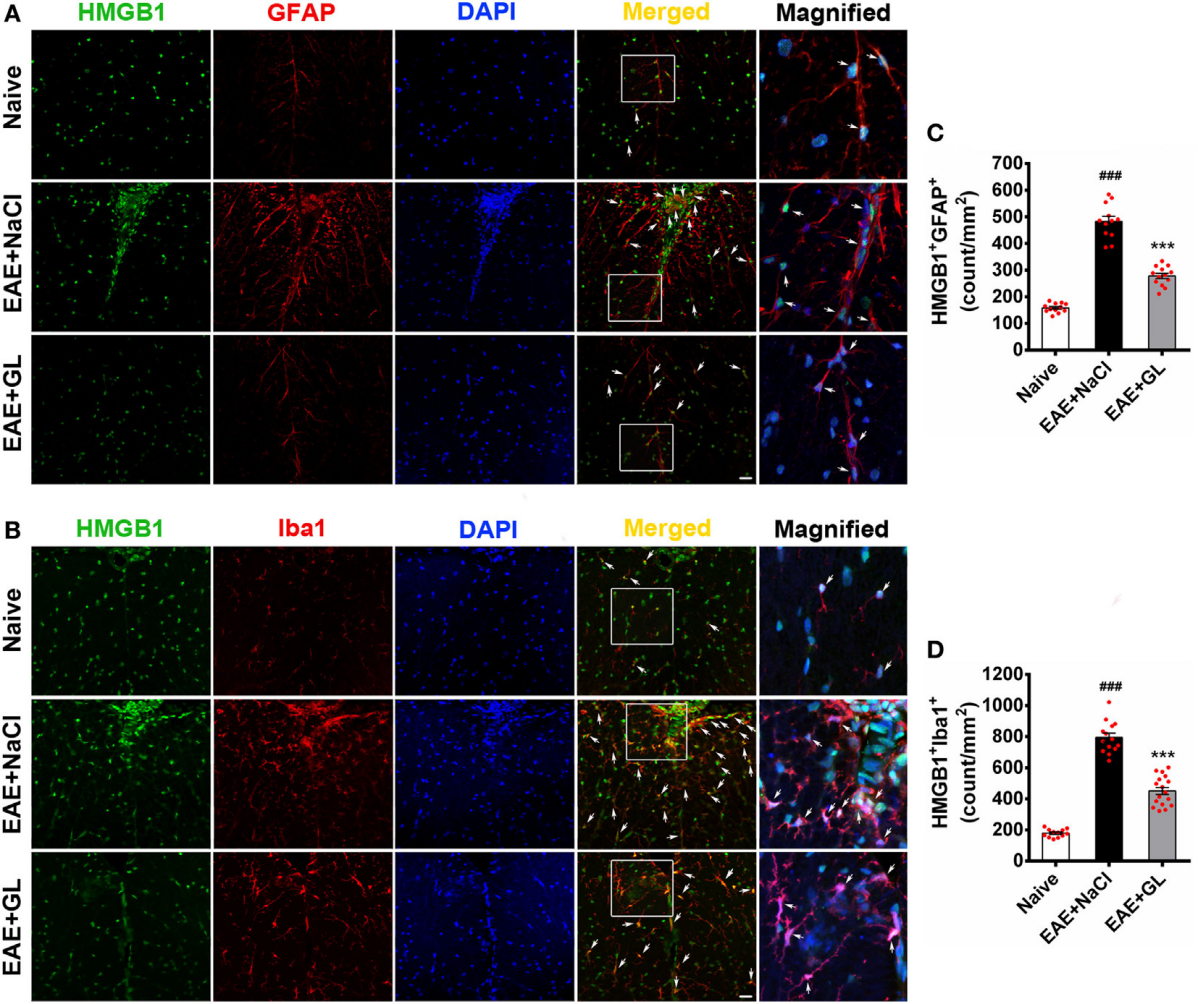
The number of high-mobility group box 1 (HMGB1)-expressing astrocytes and microglia in the thoracic spinal cord of experimental autoimmune encephalomyelitis (EAE) mice treated with glycyrrhizin (GL). HMGB1 was labeled with green fluorescence [glial fibrillary acidic protein (GFAP)] and Iba1 was labeled with red color, respectively. Nuclei were stained with DAPI in blue. **(A)** The expression of HMGB1 and GFAP in the white matter (WM) following confocal microscopy is indicated by white arrows. White boxes in the merged images are magnified regions. Arrows indicate HMGB1^+^GFAP^+^ cells. Scale bar: 20 µm. **(B)** The expression of HMGB1 and Iba1 in WM following confocal microscopy is indicated by white arrows. White boxes in the merged images are magnified regions. Arrows indicate HMGB1^+^Iba1^+^ cells. Scale bar: 20 µm. **(C,D)** The number of HMGB1^+^GFAP^+^ and HMGB1^+^Iba1^+^ cells was significantly different in GL-treated and NaCl-treated EAE mice. Data are expressed as the mean ± SEM, *n* = 4 animals in each experimental group. ^###^*P* < 0.001 vs naive group; ***P < 0.001 vs EAE + NaCl group, using one-way ANOVA followed by Bonferroni’s test.

To identify the HMGB1-expressing cells, frozen spinal cord tissue sections were used to stain for markers of various cell types. The results revealed that HMGB1 (green) protein signal co-localized with the nuclei of some GFAP^+^ astrocytes (Figure 5A, top panels) and Iba1^+^ microglia (Figure 5B, top panels) in the ventral column of the spinal cord of naive mice. Compared with naive mice, resting astrocytes and microglia became activated in EAE + NaCl mice, and HMGB1 levels significantly increased in GFAP^+^ astrocytes and Iba1^+^ microglia (Figures 5A,B, middle panel). GL administration significantly inhibited HMGB1 expression in astrocytes and microglia (Figures 5A,B, bottom panels). Moreover, the number of HMGB1^+^GFAP^+^ and HMGB1^+^Iba1^+^ cells was significantly increased in the EAE + NaCl group compared with the naive group, which was significantly reduced in mice that received GL, as shown in Figures 5C,D. These data suggest that astrocytes and microglia are important resources of HMGB1, and GL treatment reduces HMGB1 expression *via* a pathway involving both astrocytes and microglial in the spinal cord.

### GL Inhibited HMGB1 Release in Inflammation-Stimulated Neurons

**Figure 6:**
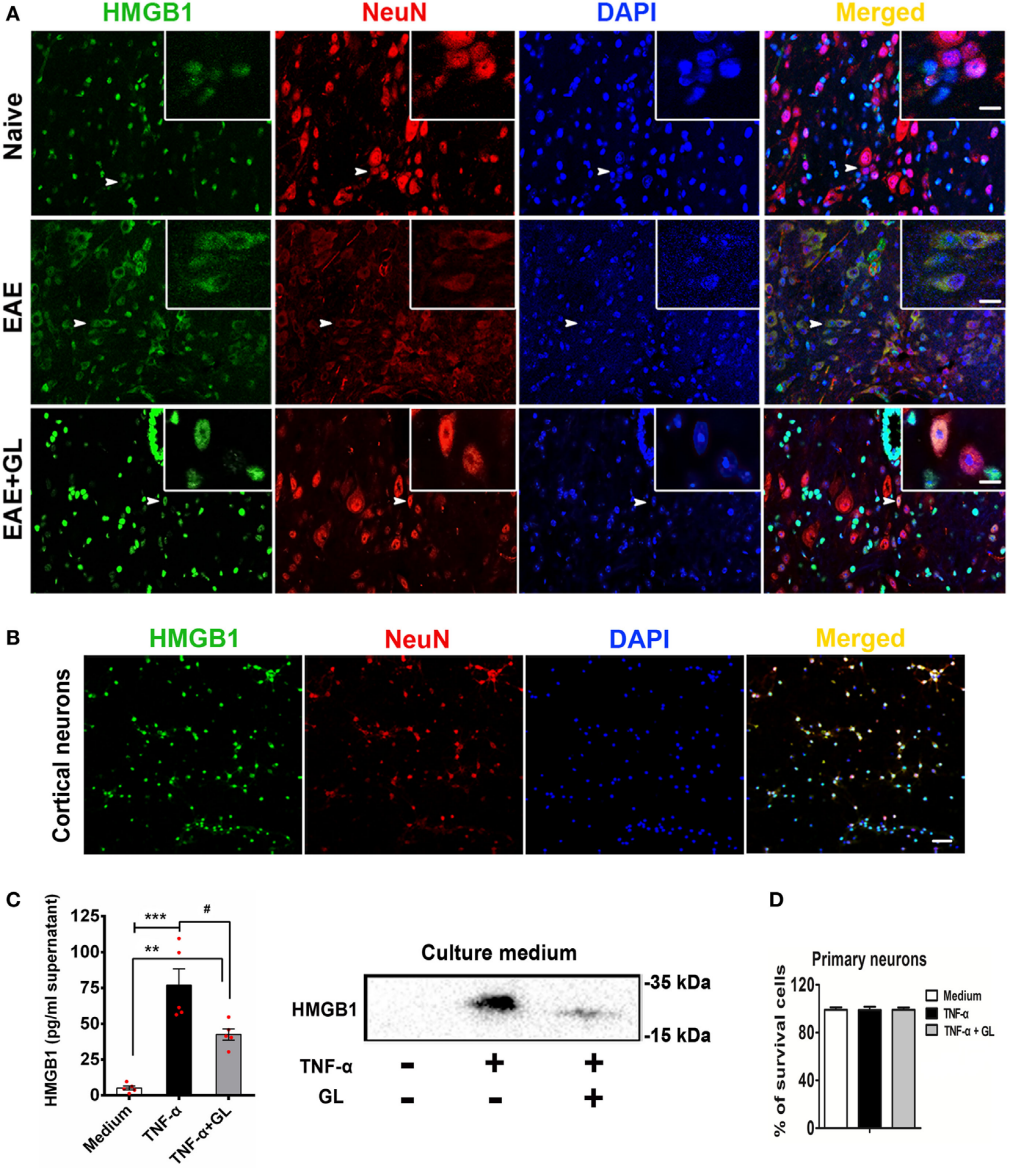
Translocation and release of high-mobility group box 1 (HMGB1) in neurons. **(A)** The translocation of HMGB1 in neurons of experimental autoimmune encephalomyelitis (EAE) mice. Representative images of DAPI (blue), HMGB1 (green), and NeuN (red) staining in the central canal of the spinal cord. Co-localization of HMGB1 and NeuN-positive cells is indicated by white arrows. White boxes in the merged images are magnified regions. *n* = 4 animals in each experimental group, and representative images are shown. Scale bars: 50 µm. **(B)** The localization of HMGB1 in primary cultured cortical neurons. Immunostaining of neurons with HMGB1 (green), NeuN (red), and DAPI (blue); representative images are shown. Scale bars: 50 µm. **(C)** ELISA and Western blotting were used to detect HMGB1 in the culture medium of primary cultured cortical neurons. Neurons were stimulated with tumor necrosis factor-alpha (TNF-α) (200 ng/mL) in the absence or presence of GL (3 mmol/L) for 18 h, and culture media were collected and concentrated using ultrafiltration tubes for subsequent ELISA and Western blot analysis. Data are shown as the mean ± SEM, *n* = 5 of each experimental group. **P < 0.01, ***P < 0.001 vs Medium group; ^#^*P* < 0.05 vs TNF-α group, using one-way ANOVA followed by Bonferroni’s test. **(D)** The survival rate of neurons was determined with Trypan blue staining after TNF-α or TNF-α + GL stimulation. The results shown are representative of three independent experiments.

We explored the effects of GL on the translocation and release of HMGB1 in EAE. As shown in Figure 6A, HMGB1 was translocated from the nucleus to the cytoplasm in central canal neurons of spinal cord in EAE mice compared to the naive mice. However, the translocation of HMGB1 was then suppressed by GL treatment. TNF has been shown to be associated with the pathophysiology of MS (34). Our data also showed that the levels of TNF-α were elevated in the CNS during EAE progression. Next, we sought to determine whether neurons released HMGB1 after TNF-α stimulation. We found that in primary cultured cortical neurons (Figure 6B), HMGB1 was released into the culture medium after TNF-α stimulation, and the release of HMGB1 was then reversed by GL administration (Figure 6C). As TNF-α and GL treatment did not induce neuronal death (Figure 6D), the above data suggest that GL inhibits HMGB1 active release from inflammation-stimulated neurons. Furthermore, as indicated in Figure S2A in Supplementary Material, we found that both neurons and BV2 cells could secret HMGB1 after TNF-α stimulation. However, neurons secreted much more HMGB1 than BV2 cells, indicating that neurons maybe the main source of HMGB1 in EAE. As expected, the release of HMGB1 was then reversed by GL administration. In addition, above treatments did not induce cells death (Figure S2B in Supplementary Material). And as indicated in Figure S3A in Supplementary Material, we cultured BV2 cells with mediums from neurons stimulated with TNF-α to investigate the effects of neuron-releasing HMGB1 on microglia and whether GL could inhibit these effects. We found that TNF-α-stimulated neuronal medium increased the secretion of HMGB1 and pro-inflammatory M1 phenotype-related cytokines, such as IL-6 and IL-1β, in the BV2 cells culture medium. The secretion of HMGB1 and other pro-inflammatory cytokines was repressed, while the anti-inflammatory M2 phenotype-related cytokines, such as TGF-β and IL-4, were increased by GL treatment (Figures S3B–D in Supplementary Material). Taken together, these results suggest that GL may play a protective role in EAE mice by inhibiting the M1 polarization of microglia, at least partly through inhibiting neuronal HMGB1 release.

## Discussion

As the therapeutic agents that are currently available to MS patients show rather limited effectiveness, novel treatment strategies are required to improve clinical outcomes. In this study, the results from *in vitro* and *in vivo* studies demonstrate that (i) HMGB1 inhibitor GL is a pivotal therapeutic agent for EAE; (ii) GL inhibits reactive glial cells and protects against neuronal damage; (iii) GL treatment exerts a protective effect against clinical symptoms, inflammation, and demyelination in EAE by inhibiting total and extracellular HMGB1 release; (iv) GL decreases HMGB1 expression in astrocytes and microglia; and (v) GL reduces active HMGB1 release in neurons after TNF-α stimulation. We found that the protective effect of GL, at least in part, is due to its inhibitory effects on HMGB1 expression and neuronal HMGB1 release during EAE.

Multiple sclerosis is an autoimmune disease of the CNS characterized by chronic inflammation, demyelination, and subsequent axonal damage, and the EAE model is a commonly used model for the study of chronic inflammation of MS. However, the potential pathomechanisms involved in disease induction and progression remain poorly elucidated. The pharmacological treatment of patients with MS is mostly based on the patient’s clinical symptoms and is not specific for the disease.

The functions of HMGB1 are dependent on its localization, and determination of the precise cellular and sub-cellular localization of HMGB1 in the CNS will help to elucidate the functions of HMGB1 in CNS homeostasis and EAE development. Extracellular HMGB1 is passively released by damaged cells or actively released by activated immune cells, including activated microglia and macrophages. HMGB1 exerts its biological functions by activating TLRs and RAGE, which in turn can promote an immune response. Many studies have shown that HMGB1 is released by neurons during subarachnoid hemorrhage and cerebral ischemia disease (19, 35). The secreted HMGB1 has been considered as a potent pro-inflammatory mediator due to the subsequent increases in levels of IL-1β and TNF-α (36), suggesting that HMGB1 induces progressive neuroinflammation when neurons are injured. In previous works, we have focused on HMGB1 expression patterns in the CNS during EAE progression, and we demonstrated an increase of extracellular and total HMGB1 in the spinal cord and found that blockade of extracellular HMGB1 with anti-HMGB1 monoclonal body could ameliorate EAE (20).

A recent study reported that GL is an HMGB1 inhibitor (37). GL can specifically bind to HMGB1 and inhibit its activity, and it was used to protect the brain by inhibiting the inflammatory response after cerebral ischemia (38). In addition, the GL derivative carbenoxolone is a widely used gap junction/hemichannel inhibitor. Suzumura and colleagues have reported that carbenoxolone treatment attenuates EAE by blocking glutamate release from microglia and delays the onset of EAE by reducing the population of Th17 cells in EAE mice (39, 40). However, these researchers did not focus on whether carbenoxolone could inhibit HMGB1. In this study, we observed the effects of GL on the development of EAE, particularly to acquire a better understanding of the mechanism of GL.

The objective of the study was to determine whether GL could serve as a unique therapeutic agent for the treatment of EAE. Thus, mice were treated using three different strategies. We found that 50 mg/kg GL treatment from days −1 to 11 (referred to as early treatment) had a short-term protective effect in EAE mice, whereas both 25 and 50 mg/kg GL treatment from days 12 to 22 (referred to as midterm-late treatment) or from days 15 to 23 (referred to as late treatment) had long-term protective effects in EAE mice. Furthermore, midterm-late treatment with 25 and 50 mg/kg GL clearly decreased the disease incidence and delayed the EAE onset time, indicating that GL treatment from the onset of EAE might confer the best efficacy. The reason for the short-term protective effect of early GL treatment could be as follows: as reported in our previous paper (20), HMGB1 shows sustained release from the early to late stage of EAE, but the inhibitory effect of GL on HMGB1 in the early stage fails to last until the late stage. However, it is surprising that early GL treatment could not delay the onset of EAE, which is inconsistent with the reports by Suzumura and colleagues showing that treatment with the GL derivative carbenoxolone from the day after immunization delayed the onset of EAE (39, 40). Further increasing the dose of GL, e.g., to 100 mg/kg, may be effective and requires further confirmation.

Moreover, we found that midterm-late GL treatment reduced demyelination and mononuclear cells infiltration in the CNS, including CD3^+^ T-cells. Th1, Th17, Th2, and regulatory T (Treg) cells play critical roles in the pathogenesis of MS/EAE, as previously reported (41, 42). HMGB1 has been reported to regulate the proliferation, activation, and infiltration of these T helper cells and Treg cells (43). Although it is not addressed here, GL treatment may affect the function and infiltration of these T cells or other immune cells induced by HMGB1 in EAE. In midterm-late GL-treated EAE mice, we also found that TNF-α, IFN-γ, IL-17A, and IL-6 levels decreased while the IL-4 level increased in serum and spinal cord homogenate. This result is consistent with previous reports showing that increased Th1/Th17 pro-inflammatory cytokines (increased levels of TNF-α, IFN-γ, and IL-17A) and decreased Th2 anti-inflammatory function (decreased levels of IL-4) are responsible for promoting MS (44). By contrast, our observation of a reduced TGF-β1 level in the serum and spinal cord homogenate of GL-treated EAE mice is somewhat surprising. Many MS/EAE treatments increase Treg cells, which have been found to be efficacious, while other treatments, although successful, have shown no effects on these cells (45). Indeed, our present data suggest that TGF-β1 is not required for the beneficial anti-inflammatory effects; however, they do not exclude the contribution of a GL treatment-induced anti-inflammatory mediator. The results also demonstrate that GL has anti-inflammatory and immunomodulatory effects in EAE and further confirm that HMGB1 is involved in the pathogenesis of this condition. Thus, increased HMGB1 levels in blood or CSF may serve as diagnostic marker. HMGB1 has more complicated physiological functions in the CNS.

Although HMGB1 has a certain degree of correlation with MS/EAE (10, 20), the role of HMGB1, especially CNS-derived HMGB1, in the MS/EAE is far less clear. In the midterm-late GL-treated mice, the significantly decreased number of HMGB1^+^ cells in the WM was likely due to the inhibition of T lymphocyte infiltration and glial cell activation by GL. Moreover, the number of HMGB1^+^ cells was not significantly reduced in the GM. Neurons are an important source of HMGB1 in the GM. It is possible that the recovery of neurons after GL treatment diminished the difference in number of HMGB1^+^ cells in the GM between the NaCl control group and GL treatment group. Thus, the decreased expression of HMGB1 in the spinal cord after GL treatment was mainly due to the decrease in WM rather than GM. In the present study, GL played a suppressive role in the stage of development rather than the initiation stage of EAE. Furthermore, GL suppressed HMGB1 expression and activation of astrocytes and microglia, and it recovered damaged neurons. Taken together, these results indicate that GL provides a novel therapeutic strategy for EAE.

Previous report has shown that HMGB1 is released by damaged neurons (19), which may contribute to the initial stages of the inflammatory response and exacerbate tissue damage. However, they do not confirm whether the release is active or passive. Whether HMGB1 is actively released by neurons is still poorly understood. As expected, we found that HMGB1 localized mostly in the cytoplasm but not the nucleus of neurons around the central canal in EAE compared with naïve mice, suggesting that HMGB1 might be translocated from the nucleus to the cytoplasm and actively released by neurons during EAE. And GL treatment suppressed the translocation of HMGB1 in neuron. Previous studies have indicated that the pro-inflammatory cytokine IL-1β leads to the release of HMGB1 from primary cortical astrocytes (18). We have reported that the levels of TNF-α are elevated in the CNS during EAE progression (29). Thus, we hypothesized that TNF-α can stimulate HMGB1 release from neuronal cells in EAE. Here, we found that TNF-α induced neurons to actively secrete HMGB1, whereas GL inhibited this release. A growing amount of information implicates HMGB1 release to be dependent on its posttranslational modifications, such as acetylation, methylation, phosphorylation, and redox effects (46). It appears that acetylation in particular position plays a vital role in the regulation of HMGB1 release (16). It has been reported that TNF-α can promote the acetylation of nuclear transcription factor (47). However, whether TNF-α promotes HMGB1 acetylation and ultimately release from neurons requires further study.

Microglia plays a dual role in mediating neuroinflammation depending on their different activation states. HMGB1 has been reported to promote the development of M1 microglia with neurotoxic and pro-inflammatory functions (48–50). As TNF-α stimulates neurons to release large amounts of HMGB1, therefore, we cultured the BV2 microglia cell line with mediums from neurons stimulated with TNF-α to investigate the effects of neuron-releasing HMGB1 on microglia and whether GL could inhibit these effects. As a result, we found that GL treatment significantly reduced the secretion of M1 phenotype-related cytokines, such as IL-6 and IL-1β, by microglia, while further enhancing the secretion of M2 phenotype-related cytokines, such as IL-4 and TGF-β1, consistent with a previous report (50). These results suggest that GL attenuates EAE at least partly by inhibiting neuronal HMGB1 release.

In summary, we showed that pharmacological inhibition of HMGB1 by employing GL has a protective effect against EAE. These results indicate that inhibition of HMGB1 expression and release can alleviate CNS demyelination and neurodegeneration after EAE. The mechanism of neuronal HMGB1 release and its signal pathway require further investigation. GL might be a promising novel tool for the treatment of human MS.

## Ethics Statement

The animal study was carried out in strict accordance with approved animal use protocols and guidelines of the Tongji Medical College Animal Care and Use Committee.

## Author Contributions

FZ worked on conception and design; YS and HC performed the majority of the experiments and analyzed the data; ZW, ZH, WC, PX, YX, and FG contributed to the experimentation; YS wrote the paper; JD provided technical support and revised the manuscript.

## Conflict of Interest Statement

The authors declare that the research was conducted in the absence of any commercial or financial relationships that could be construed as a potential conflict of interest.
