# Supplementary material for: Glycyrrhizin Protects Mice Against Experimental Autoimmune Encephalomyelitis by Inhibiting High-Mobility Group Box 1 (HMGB1) Expression and Neuronal HMGB1 Release
Source: Front Immunol. 2018 Jul 2;9:1518. doi: 10.3389/fimmu.2018.01518 (PMC6036111; doi:10.3389/fimmu.2018.01518)
Supplement: Supplementary file 1 [file presentation_1.PDF]

Supplementary Material

Glycyrrhizin Protects Mice against Experimental Autoimmune Encephalomyelitis by Inhibiting HMGB1 Expression and Neuronal HMGB1 Release

Yan Sun, Huoying Chen, Jiapei Dai, Zhongjun Wan, Ping Xiong, Yong Xu, Zhengrong Han, Weitai Chai, Feili Gong, Fang Zheng*****

*** Correspondence:** Fang Zheng: zhengfangtj@hust.edu.cn.

Supplementary Figures

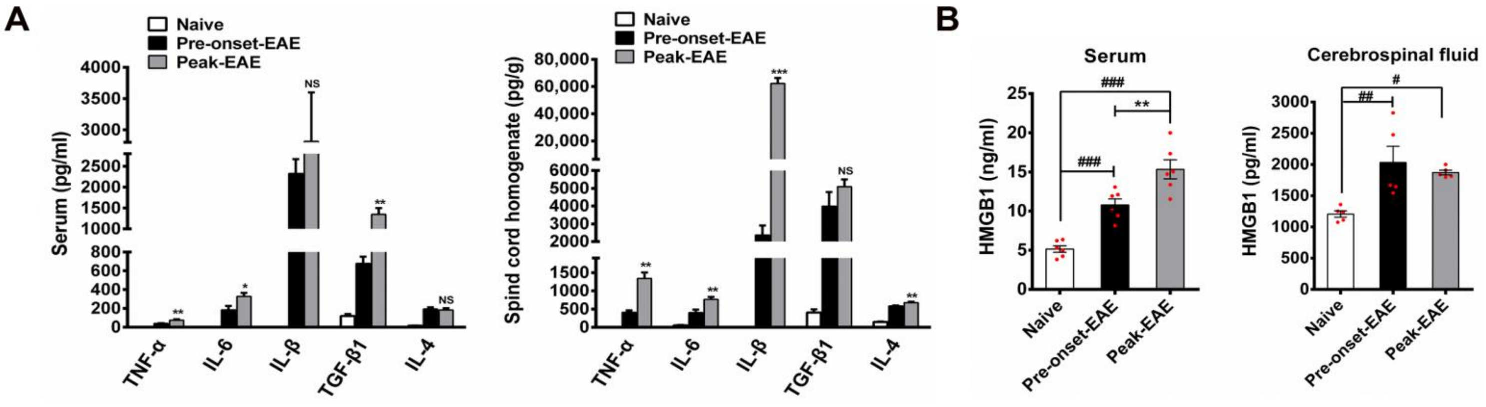

Supplementary Figure 1. The expression of inflammation-related cytokines and HMGB1 in EAE mice. The concentrations of TNF-α, IL-6, IL-1β, TGF-β1 and IL-4 **(A)** and HMGB1 **(B)** in srum, spinal cord homogenate or cerebrospinal fluid measured by ELISA from days 1 to 11 (on the day 7) and days 12 to 22 (clinical score 3) post-immunization. Data are shown as the mean ± SEM., n = 6 in each experimental group. $^{\#}P < 0.05$, $^{\#\#}P < 0.01$, $^{\#\#\#}P < 0.001$ vs Naïve group; $^{*}P < 0.05$, $^{**}P < 0.01$, $^{***}P < 0.001$ *vs* Pre-onset group, using one-way ANOVA followed by Bonferroni's test. NS: not statistically significant. TNF-α: tumor necrosis factor. TGF-β1: transforming growth factor-beta 1.

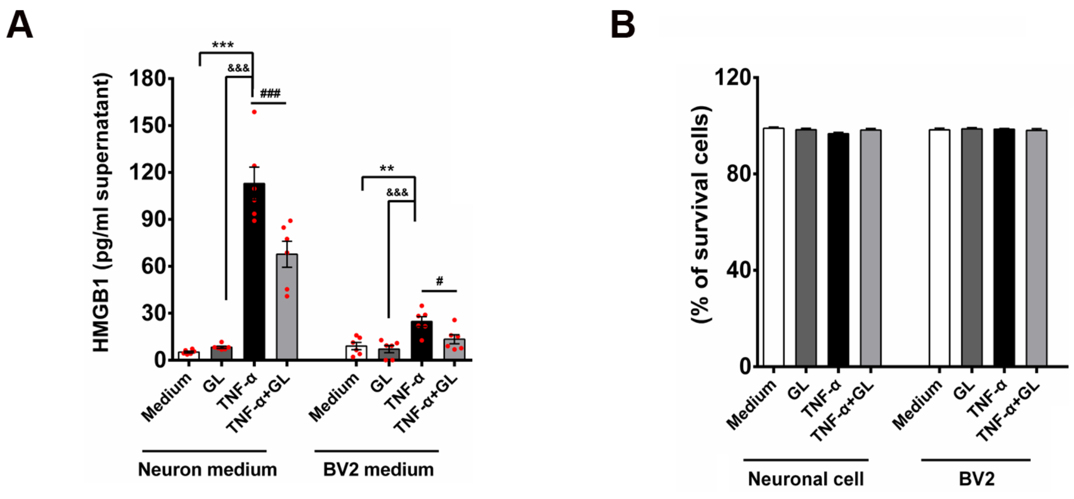

Supplementary Figure 2. The effect of GL on neurons or microglia cultured with medium from TNF-α-stimulation. **(A)** The concentration of HMGB1 in mediums was measured by ELISA after treatment. Briefly, primary cortical neurons were plated at a density of 2×10^5 cells/well in a 24-well companion plate and cultured at 37 ℃ in humidified 5% CO_2 for 7 days. Then the neurons were treated with TNF-α (200 ng/mL) in the presence or absence of GL (3 mmol/L) or vehicle treatment conditions. Meanwhile, BV2 cells stimulated with GL, TNF-α, TNF-α+GL or vehicle for 18 h. After 18 h of incubation, the mediums and cells were harvested for the following experiments. **(B)** The survival rate of neurons and BV2 cells were detected by the Trypan blue exclusion method. Data are shown as the mean ± SEM, n = 4 or 6 of each experimental group. $^{**}P < 0.01$, $^{***}P < 0.001$ vs Medium group; $^{\&\&\&}P < 0.001$ vs GL group; $^{\#}P < 0.05$, $^{\#\#\#}P < 0.001$ vs TNF-α group, using one-way ANOVA followed by Bonferroni's test.

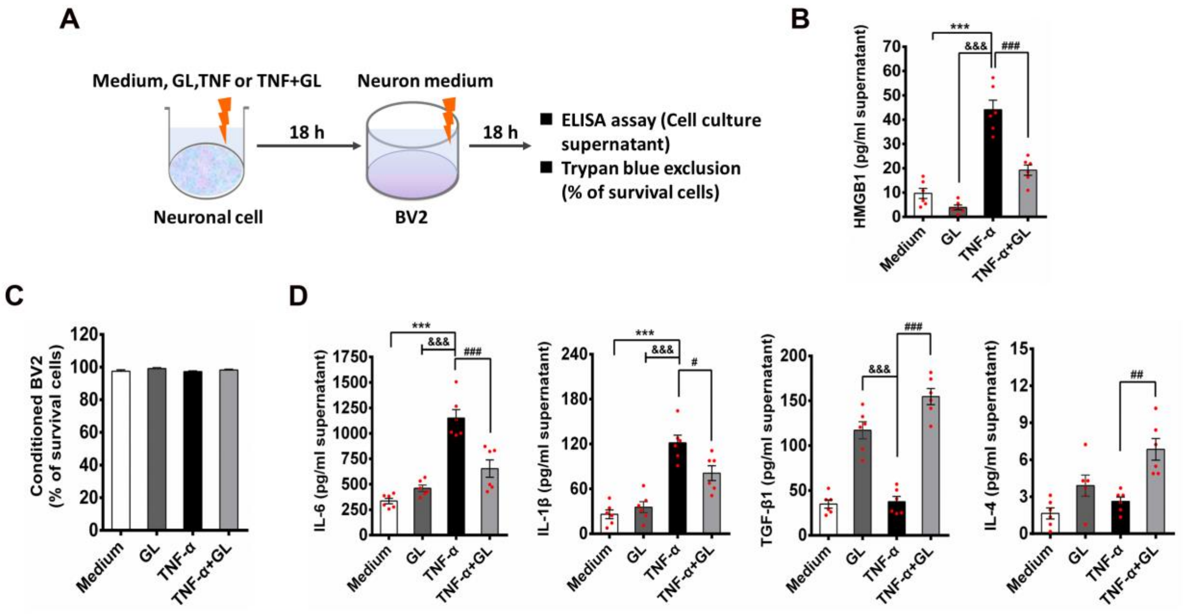

Supplementary Figure 3. The effect of GL on microglia cultured with medium from TNF-α-stimulated neurons. **(A)** Schematic workflow of the experimental design. Briefly, primary cortical neurons were plated at a density of 2 × 10^5 cells/well in a 24-well companion plate and cultured at 37 ℃ in humidified 5% CO_2 for 7 days. Then the neurons were treated with TNF-α (200 ng/mL) in the presence or absence of GL (3 mmol/L) or vehicle treatment conditions. After 18 h of incubation, the neuronal cells medium were collected and transferred into wells containing the BV2 cells which were plated at a density of 2 × 10^5 cells/well in a 24-well companion plate. After 18 h of incubation, the mediums were collected and BV2 cells were harvested for the following experiments. **(B)** The concentration of HMGB1 in mediums was measured by ELISA after treatment. **(C)** The survival rate of BV2 cells was detected by the Trypan blue exclusion method. (**D**) The concentrations of IL-6, IL-1β, TGF-β1 and IL-4 in mediums were measured by ELISA after treatment. Data are shown as the mean ± SEM, n = 6 of each experimental group. ***$P < 0.001$ vs Medium group; $^{\&\&\&}P < 0.001$ vs GL group; $^{\#}P < 0.05$, $^{\#\#}P < 0.01$, $^{\#\#\#}P < 0.001$ vs TNF-α group, using one-way ANOVA followed by Bonferroni's test.
